# The circadian timing of noise exposure influences noise-induced inflammatory responses in the mouse cochlea

**DOI:** 10.1016/j.bjorl.2021.05.010

**Published:** 2021-06-12

**Authors:** Shichang Li, Hongwei Zheng, Zhimin Xing, Yan Liu, Lin Han, Zijing Wang, Lisheng Yu

**Affiliations:** a Peking University People's Hospital, Department of Otorhinolaryngology Head and Neck Surgery, Beijing, PR China; b Bei Jing Ji Shui Tan Hospital, Department of Otorhinolaryngology Head and Neck Surgery, Beijing, PR China

**Keywords:** Noise-induced hearing loss, Circadian timing, Cochlea, Inflammation

## Abstract

**Introduction:**

Noise-induced hearing loss is one of the most common forms of sensorineural hearing loss. Nevertheless, the mechanisms of noise-induced hearing loss are still not fully understood.

**Objective:**

To investigate the dynamics of inflammatory responses in the mammalian cochlea following noise trauma at two different times, once during the light cycle and once during the dark.

**Methods:**

We challenged C57BL/6J mice with moderate, continuous noise trauma at either 9 a.m. or 9 p.m. Auditory function, histological changes in hair cells, and modifications in gene expression levels of inflammatory mediators were assessed at specific time points. Shifts in auditory brainstem response thresholds were measured at 1, 3, 7 and 14 days after noise exposure to measure potential noise-induced hearing loss. Cochlear basilar-membrane immunofluorescent staining was performed at 3 and 14 days after noise exposure. The mRNA levels of several inflammatory mediators were measured via quantitative real-time polymerase chain reaction before (pre) and after (0, 3, 12, 24 and 72 h) noise exposure.

**Results:**

We found that all noise-exposed mice developed a temporary threshold shift and that there were no significant differences between daytime and nighttime noise exposures in terms of inducing hearing-threshold shifts. Similarly, we did not detect significant histological changes in hair cells between these two groups. However, we discovered an interesting phenomenon in that the peak mRNA levels of IL-1β, IL-6, CCL2 and TNF-α were higher in day noise-exposed mice compared to those in night noise-exposed mice, and these mRNA levels subsided more slowly in day noise-exposed mice.

**Conclusion:**

Overall, these observations suggest that the circadian timing of noise exposure has a significant effect on noise-induced inflammatory responses in the mouse cochlea and that a greater inflammatory response might occur after daytime exposure.

## Highlights

• A 1-hr exposure to 6∼12 kHz noise at 100 dB SPL could cause a temporary threshold shift in C57BL/6J mice without hair cell loss.
• The circadian timing of noise exposure did not affect ABR threshold shifts.
• The circadian timing of noise exposure influences noise-induced inflammatory responses in the mouse cochlea.
• Daytime noise may induce a greater inflammatory response than that of nighttime noise.

## Introduction

Substantial efforts have been made over the years to understand the pathophysiological mechanisms underlying noise-induced cochlear injury in order to develop pharmacological interventions to reduce or prevent noise-induced hearing loss (NIHL). Recently, it was reported that mice receiving noise exposure at night suffered permanent hearing loss while the same exposure during the day resulted in only temporary hearing loss.^1^ There is emerging evidence that cochlear inflammation may be a major contributor to noise-induced cochlear injury.^2^ Several studies have demonstrated an inflammatory response in the cochlea following exposure to traumatic noise that involves an upregulation of proinflammatory mediators (e.g., cytokines, chemokines, and cell-adhesion molecules), followed by a rapid recruitment and infiltration of inflammatory cells from the systemic circulation.^3^, ^4^, ^5^ As the majority of previous studies have been performed on rodents during the daytime (their inactive period), such previous results may not provide meaningful data on how noise-induced inflammation and the circadian system interact to regulate auditory function. Thus, the purpose of the present study was to investigate the dynamics of inflammatory responses in the mammalian cochlea following noise trauma at two different times, once during the light cycle and once during the dark. In addition, we evaluated the expression of several key inflammatory markers in the cochlea including the proinflammatory cytokines, tumor necrosis factor alpha (TNF-α) and interleukin-1beta (IL-1β), as well as IL-6, chemokine (C–C motif) ligand 2 (CCL2), and glucocorticoid receptors (GRs), all of which have important roles in the recruitment and infiltration of inflammatory cells into tissues. Our study provides comparative analysis of inflammatory markers in the cochlea after exposure today and night noise In addition, we compared hearing levels and hair cell injury between day and night noise.

## Methods

### Animals

A total of 204 C57BL/6J male mice, aged 3–4 weeks, weighing 15–18 g, were used in this study, after they were confirmed to have normal hearing prior to noise exposure. Before the start of experiments, all mice were allowed to acclimatize to the animal facility for at least two weeks after delivery. Mice were housed in groups of five mice per cage on an artificial light/dark cycle (12/12 h, lights on at 0600 h, 200–400 lx at cage level), with ad libitum access to food and water. Each cage was bedded with sawdust and contained environmental enrichment (a paper nest and shredded paper). Temperature was maintained between 19°–21 °C. Because lights were on at 6 a.m. and off by 6 p.m., we set 6 a.m. as ZT0. Handling at ZT 12–24 (darkness) was performed under dim red light. All mice were purchased from Vital River Laboratory Animal Technology Co. Ltd. (Beijing, China). Care and use of the animals in this study was approved by the Institutional Animal Care and Use Committee (IACUC) of the Peking University Peope’s Hospital (2019PHE072).

### Experimental design

**Figure 1:**
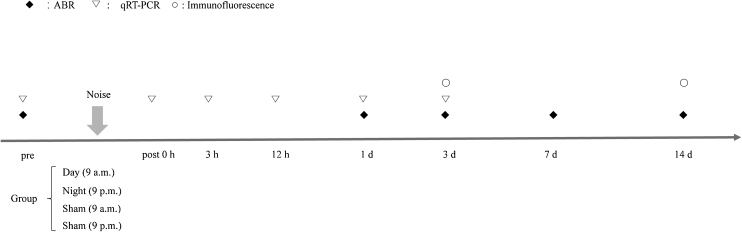
Schematic of the experimental design. Auditory brainstem response (ABR) thresholds were measured at five time points: prior to and at 1, 3, 7 and 14 days after noise exposure.

Healthy mice were randomly divided into four groups: (i) daytime noise (9 a.m.); (ii) daytime sham (9 a.m.); (iii) nighttime noise (9 p.m.); and (iv) nighttime sham (9 p.m.). Zeitgeber time 0 (ZT0) denotes the initiation of lights on (i.e., 0 h in ZT) and the start of the inactive phase, while ZT12 denotes lights off (i.e., 12 h after the ZT0 initiation of lights on) and the start of the active phase. Mice in the daytime noise group were exposed to noise from 9 a.m. to 10 a.m. (day, ZT3–4), whereas mice in the nighttime noise group were exposed to noise from 9 p.m. to 10 p.m. (night, ZT15–16). The sham controls for daytime and nighttime noise were placed in an acoustic chamber in mesh cages for 1 h at 9 a.m. and 9 p.m., respectively, but no acoustic stimulation was delivered. These two time points were selected because they correspond to the peak and trough levels of cochlear *per2* Protein Expression (PER2), which are slightly delayed from peak (ZT12) and trough (ZT0) *per2* mRNA levels.^1^ The experimental mice were used for time-course studies at 0, 3 and 12 h, as well as 1-, 3-, 7- and 14-days following noise exposure. The schedule of the experiments is shown schematically in Fig. 1.

### Auditory brainstem responses (ABRs)

Electrophysiological measurements were made in a double-walled soundproof room. Prior to noise exposure, subjects underwent testing of baseline auditory brainstem responses (ABRs) in their right ears. The mice were anesthetized with a mixture of gaseous isoflurane (2.5% for induction, 1.2% for maintenance, body temperature maintained at ∼37 °C) and oxygen (1 L/min flow rate) from an oxygen concentrator (Pureline OC4000, Supera, Clackamas, OR). Testing was performed in a sound-attenuating booth. Three 6-mm platinum electrodes (Rochester Electro-Medical, Lutz, FL) were inserted subdermally. The reference electrode was inserted beneath the pinna of the right ear, the ground beneath the lift ear, and the active electrode beneath the skin at the vertex. The stimuli were generated using Tucker Davis Technologies (TDT, Gainesville, FL) SigGen software. Each tone burst was 1-ms in duration and had a 0.5-ms rise/fall time with no plateau. Stimuli were presented at a rate of 19 stimuli/s. Acoustic stimuli were delivered monaurally to a Beyer earphone attached to a customized plastic speculum inserted into the ear canal. The evoked responses of the mice were amplified with a gain of 50,000, using a TDT RA4LI head stage connected to an RA4PA preamplifier, and were band-pass filtered from 0.1 to 3.0 kHz. Frequencies between 4.0–32 kHz, as well as click sounds, were tested at various levels from 90 to 10 dB SPL in 5-dB decrements. Additionally, 1024 sweeps were averaged at each stimulus level using TDT BioSigRz software. The ABR waveform of each mouse consisted of primarily two positive peaks followed by two negative troughs. The second positive-negative complex typically had the larger amplitude of the two and was the wave that was tracked to determine thresholds. Thresholds were defined as the lowest sound pressure level at which a repeatable response could be detected. During all ABR analyses, the evaluator was blind to the experimental condition for each mouse. The hearing level of each mouse was measured before noise exposure, and at 1-, 3-, 7- and 14-days after noise exposure. Hearing of mice in the daytime noise group and sham group was measured at 10 a.m., whereas hearing of mice in the nighttime noise group and sham group was measured at 10 p.m. Hearing thresholds are reported as means ± Standard Errors of the Means (SEM).

### Noise exposure

Mice were exposed to a narrow-band noise centered at 6–12 kHz for 1 h continuously at an intensity of 100 decibels (dB) sound pressure level (SPL) as in the study of Meltser et al.^1^ Alert, non-anesthetized mice were placed in a special restraint cage and were ensured that each ear received noise exposure of equal intensity. Each mouse was exposed to noise in a separate individual cage to prevent any shielding from the noise and to ensure as uniform of a noise dose across mice as possible. During the exposure, the mice did not have any access to food or water. The sound exposure chamber was fitted with a loudspeaker (YH25-19B, 25 W, 16 Ω, China) driven by a power producer (33220 A, China) fed from noise software. The noise sound files were created and equalized with audio-editing software (Audition 3; Adobe System, Inc., San Jose, CA). Sound levels were calibrated with a sound-level meter (model 1200; Quest Technologies, Oconomovoc, WI) at multiple locations within the sound chamber to ensure uniformity of the sound field. All sampled noise for calibration was within ±1 dB of the 100 dB SPL level. Sound levels were measured before and after exposure to ensure stability.

### Assessment of sensory hair-cell damage

Cochlear tissues were harvested following different experimental paradigms. Mice were sacrificed by decapitation under deep isoflurane anesthesia. The cochleae were quickly removed from the skull. The round and oval windows and the apex of the cochlea were opened, through which the cochlea was perfused with 4% paraformaldehyde and fixed overnight. After fixation, the cochlear shell was decalcified with 10% EDTA for 4–6 h, and the basal turn was separated under a dissection microscope in 0.01 mmoL/L of phosphate-buffered saline (PBS). The cochleae were dissected by removing the lateral wall bones and tissues, as well as the tectorial membranes. After washing several times with PBS, the remaining parts of the cochleae were incubated in 0.3% Triton X-100 in PBS for 5 min, after which they were washed three times with PBS. Immunolabeling was carried out overnight at 4 °C with a rabbit polyclonal cleaved caspase-3 antibody (1:100; catalog #9661, Cell Signaling Technology, Danvers, MA, USA). Caspase-3 is an important member of the caspase family, as it participates in the execution of disassembly of apoptotic cells.^6^ Furthermore, the activation of caspase-3 has been regarded as a hallmark of apoptosis.^7^ The sections were rinsed in PBS and were subsequently incubated with a goat anti-rabbit IgG (H + L), F(ab′)_2_ fragment (Alexa Fluor 488 conjugate, Cell Signaling Technology, #4412) for 1 h at room temperature. After washing three times with PBS, the organ of Corti was stained for F-actin with Alexa Fluor 555 Phalloidin (Cell Signaling USA, catalog #8953) for 60 min to outline the hair cells and their stereocilia. Following three washes with PBS, the organ of Corti was dissected and mounted on slides containing an anti-fade medium (Vectashield with DAPI; Vector Laboratories, Burlingame, CA, USA). The cochlea was divided into apical (percent distance from the apex, 0.0%–33.3%), middle (33.3%–66.6%), and basal (66.6%–100.0%) turns.^8^ The number of missing OHCs and immunoreactivities for cleaved caspase-3 were counted in each turn and the percentage of missing or undergoing apoptotic OHCs was recorded. The tissues were observed under a laser-scanning confocal microscope (LSM 710; Zeiss, Germany).

### Quantitative real-time polymerase chain reaction (qRT-PCR)

To compare the inflammatory responses between groups, mice were sacrificed at 0-, 3- and 12 h, as well as at 1- and 3-days after noise exposure. Quantitative real-time polymerase chain reaction (qRT-PCR) was conducted to measure the mRNA levels of IL-1β, IL-6, TNF-*α*, CCL2, and GRs, as indicators of the inflammatory response. The whole cochlea was dissected from the temporal bone of euthanized mice, quickly frozen in liquid nitrogen. Total RNA was isolated from the mouse cochlea via commercial kit (Recover All Total Nucleic Acid Isolation Kit; Ambion, Austin, USA), according to the manufacture’s recommendations. Total RNA was quantified by measuring the absorbance ratios at 260/280 nm. Complementary DNA (cDNA) was synthesized using a high-capacity cDNA reverse transcription kit (Thermo Fisher Scientific, Waltham, MA USA). RT-PCR analysis was performed by a StepOnePlus system (Applied Biosystems, Foster City, CA, USA) using the SYBR-Green Real-Time PCR Master Mix kit (Toyobo, Osaka, Japan). Primer sequences are listed in Supplementary Material, Table 1. The relative expression levels of target genes were calculated by the 2^−ΔΔCT^ method using β-actin as the internal reference.

### Statistical analysis

For ABR, a two-way repeated-measures analysis of variance (ANOVA) coded for group (daytime or nighttime noise exposure) and time (pre, 1d, 3d, 7d, and 14d) was used. Two-way ANOVA coded for group (daytime or nighttime noise exposure) and time (pre, 0 h, 3 h, 12 h, 1d, and 3d) were used for qRT-PCR data. Bonferroni post-hoc tests were used for post-hoc pairwise comparisons when appropriate. A *p*-value of <0.05 was considered statistically significant. All tests were performed using SPSS statistical software (SPSS, Version 21.0, Chicago, IL, USA).

## Results

### Threshold shifts after noise exposure

**Figure 2:**
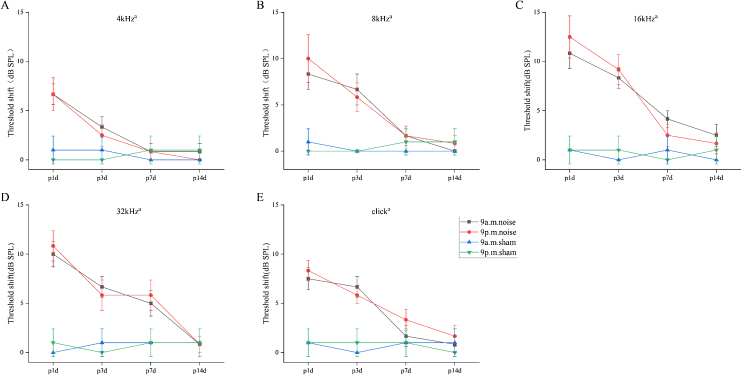
Shifts in auditory brainstem response (ABR) thresholds after noise exposure.

To evaluate how noise exposure changed the hearing thresholds in different groups, the ABR thresholds at 4, 8, 16 and 32 kHz, as well as for click sounds, were measured at the following four time points: before noise exposure (baseline or pre), and at 1-, 3-, 7- and 14-days after noise exposure. As shown in Fig. 2A—E, for all frequencies and click sound stimuli, there was a significant main effect of time, while more importantly for the purpose of the experiment, there was no main effect of group or interaction involving group (a, main effect of time, *p* < 0.05; for all frequencies and click sounds, two-way repeated-measures ANOVA). The two groups exhibited elevated hearing thresholds at 1-day after noise exposure, followed by recovery to near-baseline hearing levels over the following 2-weeks. In sham mice, hearing thresholds remained in the normal range throughout all testing days. Collectively, these findings indicated that there were no significant differences between the two different circadian noise-exposure times in terms of shifts in hearing thresholds.

### Histological assessment of noise-induced cochlear damage

To examine whether noise trauma induced hair-cell loss or apoptosis in the cochlea, whole-mount preparations of the auditory epithelium were stained with cleaved caspase-3 antibody, phalloidin, and DAPI at 3-days and 14-days after noise exposure. Three types of OHC pathologies – apoptosis, necrosis, and missing OHCs – were documented with the following criteria.^9^ Apoptotic OHCs were defined as OHCs showing condensed nuclei and positive caspase-3 staining. Necrotic OHCs were defined as OHCs showing nuclear swelling and the absence of caspase-3 staining. Missing OHCs were defined as areas of the cuticular plates without F-actin labeling and with an absence of nuclei. Hair cells appeared normal in all groups in terms of numbers and shapes, and no positively labeled cells with cleaved caspase-3 antibody were observed in any examined cochleae (data not shown).

### Changes in the mRNA levels of proinflammatory mediators and GRs

**Figure 3:**
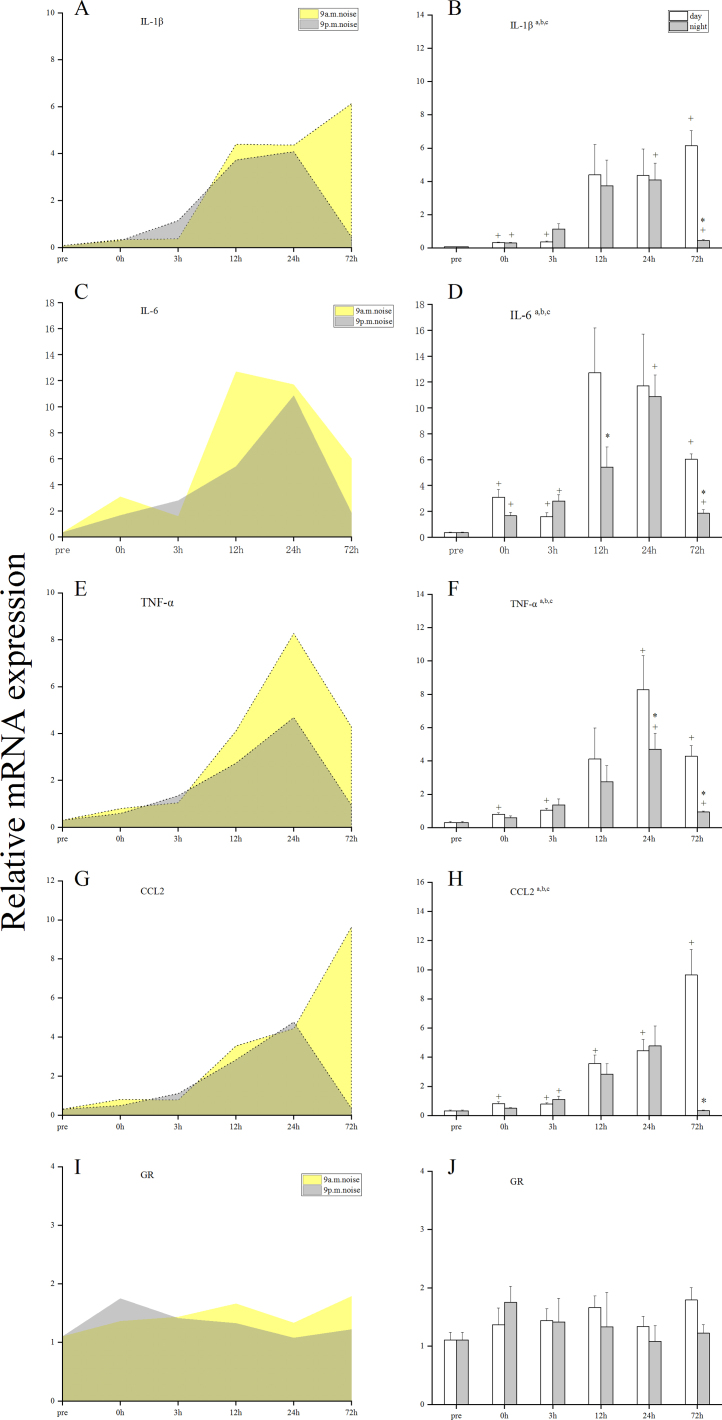
Proinflammatory mediators before and after noise exposure.

To evaluate the inflammatory status in noise-exposed cochleae, the mRNA levels of pro-inflammatory cytokines (TNF-α, IL-1β, and IL-6), chemokines (CCL2), and GRs at pre, 0, 3, 12, 24, and 72 h after noise trauma were analyzed by qRT-PCR. In the two sham groups, the mRNA levels of proinflammatory mediators were weak, and there were no statistically significant differences between the daytime sham group and the nighttime sham group across all time points (Supplementary Material, Fig. 1). Fig. 3 shows that the mRNA levels of IL-1β, IL-6, TNF-α, CCL2, and GRs were significantly increased after noise trauma (a, main effect of time, *p* < 0.05; b, main effect of group [day, night], *p* < 0.05; and c, interaction, *p* <  0.05). Compared with those in nighttime noise-exposed mice, the expression levels of IL-1β, IL-6, CCL2 and TNF-α were higher in daytime noise-exposed mice at 72h after noise trauma. The peak mRNA levels of IL-1β, IL-6, CCL2 and TNF-α were higher in daytime noise-exposed mice. There was no statistical difference in the expression of GR between the two different circadian noise-exposure times. Taken together, daytime noise might have a greater impact on the expression of several genes involved in proinflammatory responses. Overall, these observations suggest that nighttime noise may induce a milder inflammatory response than that of daytime noise.

## Discussion

The present study was undertaken to investigate the circadian effects of NIHL in C57BL/6J mice. Two groups of C57BL/6J mice were exposed to 100 dB SPL of 6–12 kHz noise for 1 h at either 9 a.m. or 9 p.m. ABR thresholds were measured at days 1, 3, 7 and 14 after noise exposure. The two groups of mice all developed a Temporary Threshold Shift (TTS), and analysis of ABR threshold shifts revealed no significant differences between the daytime and nighttime noise-exposure groups. At the same time, there was almost no missing, necrotic, or apoptotic OHCs in the cochleae of the two groups. Interestingly, when we measured the mRNA levels of several inflammatory mediators, we found that the daytime noise-exposure group had higher mRNA levels of IL-6, TNF-α, CCL2 and IL-1β.

Meltser et al.^1^ kept CBA/CaJ mice in a light-controlled vivarium and exposed them to continuous noise at 100 dB SPL for 1 h at two different times, once during the light cycle and once during the dark cycle. At 24 h after the exposures, both groups showed similar ABR threshold shifts. However, at two weeks after the exposures, the thresholds of the group exposed during the light cycle had fully recovered, whereas the group exposed during the dark cycle exhibited a permanent threshold shift. These findings indicate that the CBA/CaJ mouse strain has variable susceptibility to permanent NIHL depending on the time of exposure during the light/dark cycle. However, Harrison et al. found that there was only little evidence for a chrono-tolerance effect for impulse noise exposure (500 clicks at one click/sec at a level of 137 dB peSPL) in C57BL/6 J mice.^10^ For example, the ABR-threshold shifts after the impulse exposure revealed no significant differences between day and night groups at any of the test days. Kim et al. found that male BALB/c mice (four weeks old) exposed to continuous noise (300–10,000 Hz, 120 dB SPL, 3 h) during the inactive phase showed more severe hearing loss than did mice exposed to noise during the active phase.^11^ In the present study, we challenged C57BL/6J mice with moderate, continuous noise trauma (6–12 kHz, 100 dB SPL, 1 h) at either 9 a.m. or 9 p.m., corresponding to rest and active phases, respectively. We found that there were no significant differences between the two exposure times for hearing threshold shifts at any of the test days in our present study. There are several reasons that may have contributed to this discrepancy. First, Harrison et al. and our present study each used a C57BL/6J mouse strain, whereas Kim et al. used a BALB/c mouse strain and Meltser et al. used a CBA/CaJ strain. These different strains may have yielded different results, as it has been demonstrated that genetic background may modulate auditory sensitivity to noise trauma.^12^ In addition to strain differences, the mice used in Harrison et al. and our study were 4–6 weeks old, while those in Kim et al. were four weeks and those in Meltser et al. were 8–16 weeks old. Therefore, these differences in age may have induced differential effects on chrono-tolerance across these studies. Moreover, the noise exposures used in these studies were different. Importantly, it is well known that the mechanisms of cochlear damage induced by different types of noise are not all the same.

Recently, inflammation has been increasingly recognized as an important pathophysiological mechanism underlying irreparable or reparable damage to hair cells and neurons after acoustic injury.^13^ The inflammatory response involves an upregulation of inflammatory mediators generated primarily by various resident cochlear cells, followed by the rapid recruitment and infiltration of inflammatory cells from the endolymphatic sac^14^ or from the systemic circulation through the spiral modiolar vein.^15^ This noise-induced, inflammatory-related reaction may be implicated in regulating the initiation and progression of NIHL.^16^ A growing body of evidence supports a key role for the circadian clock in the regulation of immune functions and inflammatory responses.^17^ In our present study, we found that the daytime noise-exposure group had higher mRNA levels of IL-6, TNF-α, CCL2 and IL-1β. The mRNA levels of CCL2 and IL-6 was higher in animals showing permanent hearing impairment than in those showing temporary hearing impairment, suggesting that these inflammatory responses may be detrimental to hearing recovery.^18^ Overall, these observations suggest that a greater inflammatory response might occur after daytime exposure. However, there was no statistical difference in the expression of GR between the two groups and we did not find missing, necrotic, or caspase-3-positive hair cells in the cochleae of either the daytime or nighttime noise-exposure groups at 3d and 14d after noise exposure. A noise-induced downregulation of GR mRNA has been reported in several previous studies, in which the noise intensity used was higher than our experiment.^19^, ^20^, ^21^ Hence, it is possible that the influence of circadian rhythms on inflammatory responses after moderate acoustic trauma are not sufficient to affect threshold sensitivities and cause morphological damage to cochlear hair cells.

The present results indicated that noise trauma might induce a greater inflammatory response when challenged during the inactive phase in mice, which suggests that mice are less susceptible to noise trauma during the active phase. From an evolutionary standpoint, chrono-tolerance was likely designed as an anticipatory protective mechanism from environmental threats that are likely to be encountered during the active period. In rodents, the active period occurs during the dark phase, whereas the active period of humans occurs during the light phase. Therefore, the present findings indicate that the human cochlea might be more susceptible to noise exposure during the nighttime, and that hearing protection should be emphasized during this time.

Finally, our present study also had some limitations. We speculate that the inflammatory response resulting from a moderate acoustic trauma might not have reached a sufficient amplitude to cause morphological and functional changes. Hence, it may be necessary to challenge mice with a more intense acoustic trauma or repeated moderate noise levels. We believe that the lack of food or water during noise exposure time, as a sign of stress-related condition, had no impact on inflammatory response, since there was no statistical difference in the mRNA levels of inflammatory factors between the two sham groups. While, in the sham groups, adding other stimuli during the 1 h of ‘noise exposure’ to prevent the mice from sleeping will be more interesting, so as to better control the variables. In addition, the observation times in our study were relatively short, and we did not explore the diurnal sensitivity to noise trauma from the perspectives of repair and reconstruction. Hence, we plan to more comprehensively observe mice across the circadian cycle in future studies investigating circadian influences of NIHL and related inflammatory responses.

## Conclusions

Our findings suggest that the circadian timing of noise exposure had a significant effect on noise-induced inflammatory responses in the mouse cochlea and that a greater inflammatory response might occur after daytime noise exposure. As the majority of previous studies have been performed on rodents during the daytime (their inactive period), such previous results may not provide meaningful data on how noise-induced inflammation and the clock system interact to regulate auditory function. Consequently, experiments performed around the clock on rodents may provide a better understanding of the underlying mechanisms in NIHL and other auditory disorders and may open novel avenues for therapeutic approaches based on chrono-pharmacology.

## Conflicts of interest

The authors declare no conflicts of interest.
