# Supplementary material for: The circadian timing of noise exposure influences noise-induced inflammatory responses in the mouse cochlea
Source: Braz J Otorhinolaryngol. 2021 Jun 12;88(Suppl 3):S1–8. doi: 10.1016/j.bjorl.2021.05.010 (PMC9760997; doi:10.1016/j.bjorl.2021.05.010)
Supplement: Supplementary file 1 [file mmc1.docx]

**BJORL-D-20-01019 - Supplementary Material**

**Figure 1.** qRT-PCR of mRNA levels of inflammatory mediators in the sham control. There were no significant differences between the two sham groups.





**Table 1** Primers for RT-PCR.

| **Primer** | **Sequence** |
| --- | --- |
| IL-1 β-F | GCAACTGTTCCTGAACTCAACT |
| IL-1 β-R | ATCTTTTGGGGTCCGTCAACT |
| IL-6-F | TAGTCCTTCCTACCCCAATTTCC |
| IL-6-R | TTGGTCCTTAGCCACTCCTTC |
| TNF- α-F | CCTGTAGCCCACGTCGTAG |
| TNF- α-R | GGGAGTAGACAAGGTACAACCC |
| CCL2-F | TTAAAAACCTGGATCGGAACCAA |
| CCL2-R | GCATTAGCTTCAGATTTACGGGT |
| GR-F | CCCAAGAGTTCAACACCTGC |
| GR-R | AAACTCCTTCTCTGTCGGGG |
| β-actin-F | GGCTGTATTCCCCTCCATCG |
| β-actin-R | CCAGTTGGTAACAATGCCATGT |

F, Forward primer; R, Reverse primer.
